# The Passage of Microbes through the Skin

**Published:** 1893-05

**Authors:** 


### The Passage of Microbes Through the Skin.

Wasmuth describes experiments made both upon lower animals and upon man to determine the extent to which different micro-organisms can penetrate the skin. In man the pure culture of staphylococcus was rubbed into the arm; in animals, the cocci of erysipelas and anthrax. If the cultures were merely spread upon the skin, negative results were produced. By rubbing, it was found that the uninjured skin was permeable to these organisms, and that after they were introduced in this way, characteristic local and general disturbances were produced. The bacilli enter between the sheath and the shaft of hairs and not through the openings of the sweat or of the sebaceous glands.—*Boston Med. and Surg. Journal.*

